# Deletion of Cardiomyocyte Glycogen Synthase Kinase-3 Beta (GSK-3β) Improves Systemic Glucose Tolerance with Maintained Heart Function in Established Obesity

**DOI:** 10.3390/cells9051120

**Published:** 2020-04-30

**Authors:** Manisha Gupte, Prachi Umbarkar, Anand Prakash Singh, Qinkun Zhang, Sultan Tousif, Hind Lal

**Affiliations:** 1 Division of Cardiovascular Medicine, Vanderbilt University Medical Center, Nashville, TN 37232, USA; guptem@apsu.edu (M.G.); pumbarkar@uabmc.edu (P.U.); apsingh@uabmc.edu (A.P.S.); qzhang@uabmc.edu (Q.Z.); 2 Department of Biology, Austin Peay State University, Clarksville, TN 37044, USA; 3 Division of Cardiovascular Disease, UAB|University of Alabama at Birmingham, Birmingham, AL 35294-1913, USA; sana80@uab.edu

**Keywords:** GSK-3, high fat diet, obesity, cardiac function

## Abstract

Obesity is an independent risk factor for cardiovascular diseases (CVD), including heart failure. Thus, there is an urgent need to understand the molecular mechanism of obesity-associated cardiac dysfunction. We recently reported the critical role of cardiomyocyte (CM) Glycogen Synthase Kinase-3 beta (GSK-3β) in cardiac dysfunction associated with a developing obesity model (deletion of CM-GSK-3β prior to obesity). In the present study, we investigated the role of CM-GSK-3β in a clinically more relevant model of established obesity (deletion of CM-GSK-3β after established obesity). CM-GSK-3β knockout (GSK-3β^fl/flCre+/−^) and controls (GSK-3β^fl/flCre−/−^) mice were subjected to a high-fat diet (HFD) in order to establish obesity. After 12 weeks of HFD treatment, all mice received tamoxifen injections for five consecutive days to delete GSK-3β specifically in CMs and continued on the HFD for a total period of 55 weeks. To our complete surprise, CM-GSK-3β knockout (KO) animals exhibited a globally improved glucose tolerance and maintained normal cardiac function. Mechanistically, in stark contrast to the developing obesity model, deleting CM-GSK-3β in obese animals did not adversely affect the GSK-3αS21 phosphorylation (activity) and maintained canonical β-catenin degradation pathway and cardiac function. As several GSK-3 inhibitors are in the trial to treat various chronic conditions, including metabolic diseases, these findings have important clinical implications. Specifically, our results provide critical pre-clinical data regarding the safety of GSK-3 inhibition in obese patients.

## 1. Introduction

One-third of the US adults are obese, leading to an increase in obesity-associated pathologies such as cardiovascular diseases and diabetes [1,2]. Importantly, numerous studies have shown a strong association of obesity with dysregulated signaling in metabolically important tissues, such as adipose, liver, and skeletal muscle [3]. One of the organs that is recently being investigated for its role in obesity-associated cardio-metabolic dysfunction is the heart, which is not a surprise since it is well known to harbor numerous molecules shown to be critical for global metabolism e.g., natriuretic peptides, atrial natriuretic peptide (ANP), and BNP [4,5,6]. One such molecule is Glycogen Synthase Kinase-3 (GSK-3), a serine-threonine kinase initially identified for its role in glycogen synthesis [7]. GSK-3 has two ubiquitously expressed isoforms, alpha (α) and beta (β) [7,8].

Interestingly, the role and regulation of the two GSK-3 isoforms in the heart are context-dependent [9,10,11,12,13,14]. Additionally, we (and others) have demonstrated that there is a distinct tissue-specific role for GSK-3β in metabolism and cardiac pathophysiology [10,12,15,16]. Conditional deletion of GSK-3β from skeletal muscle improved glucose tolerance and insulin sensitivity [15]. In contrast, deletion of GSK-3β from the liver did not affect glucose homeostasis [15]. Interestingly, deletion of GSK-3β from insulin-producing β-cells improved glucose tolerance and increased beta-cell mass in mice fed normal as well as high-fat diet (HFD) [17] while increased hypothalamic GSK-3β led to glucose intolerance and weight gain in leptin-deficient (ob/ob) mice [18]. We reported that conditional deletion of cardiomyocyte (CM)-GSK-3β protects against myocardial infarction (MI)-induced cardiac remodeling, with no role in Transverse Aortic Constriction (TAC)-induced pathological hypertrophy and cardiac dysfunction [10]. Recently, we have shown that CM-GSK-3β is critical to cardiac function in high-fat diet-induced obesity [6,19]. CM-GSK-3β was conditionally deleted in adult mice, followed by chronic high-fat feeding for 55 weeks. At the end of the study, obese cardiac GSK-3β deficient mice exhibited modest but significant cardiac dysfunction compared to the littermate controls [6]. In the present study, we sought to determine the role of CM-GSK-3β in a clinically more relevant model where GSK-3β was deleted after establishing obesity. Interestingly, findings from this study indicate that the timing of CM-GSK-3β deletion (before or after the development of obesity) is critical to the phenotypic outcome. Specifically, in contrast to the developing obesity model [6], CM-GSK-3β deletion after established obesity did not affect cardiac function and led to an improved glucose tolerance phenotype. These findings provide crucial pre-clinical data regarding the safety of GSK-3 inhibition in obese patients.

## 2. Materials and Methods

### 2.1. Mice and HFD Treatment

CM-specific GSK-3β-KO were generated by crossing GSK-3β^fl/fl^ mice with mice carrying the Mer-Cre-Mer transgene driven by the α-myosin heavy chain promoter (a gift from Dr. J. Molkentin, Cincinnati Children’s Hospital, Cincinnati, OH, USA) [6,10]. Mice were crossed for two generations in order to generate GSK-3β^fl/flCre+/−^ mice. Both mouse strains were on the C57BL/6 background. At 10 weeks of age, when physiological development is largely complete, both GSK-3β^fl/flCre+/−^ and GSK-3β^fl/flCre−/−^ controls were subjected to control diet (CD; 10% kcal as fat; D12450K)) or HFD (60% kcal as fat; D12492) from Research Diets in order to establish obesity. Diets provided energy at 3.85 (CD) or 5.24 (HFD) kcal/g and were matched for protein content (20% kcal) and a similar source of dietary fat (lard). Diets were provided ad libitum to mice. After 12 weeks of CD or HFD, all mice received Tamoxifen (Tam) injections (20 mg/kg/day, intraperitoneal injection, IP) for five consecutive days to delete GSK-3β specifically in CMs and continued on CD or HFD for a total period of 55 weeks. GSK-3β^fl/flCre+/−/Tam^ mice were the conditional knockout (KO), whereas littermates GSK-3β^fl/flCre−/−Tam^ represented controls (WT). The Institutional Animal Care and Use Committee of Vanderbilt University Medical Center approved all animal procedures and treatments (protocol # M1700133-00). All animals were housed in a temperature-controlled room with a 12:12h light–dark cycle and received humane care.

### 2.2. Body Composition

Lean and fat mass were measured in conscious mice using the Minispec Model mq7.5 (Bruker Instruments, Billerica, MA, USA).

### 2.3. Echocardiography

Echocardiography was performed as described previously [6]. In brief, transthoracic two-dimensional motion-mode echocardiography was performed with a 12-mHz probe (VisualSonics, Ontario, Canada) on mice anesthetized by inhalation of isoflurane (1–1.5%) at 0 and 55 weeks post-HFD. Left ventricle end-systolic interior dimension (LVIDs), Left ventricle end-diastolic interior dimension (LVIDd), ejection fraction (EF), and fractional shortening (FS) values were analyzed using the Vevo2100 program (VisualSonics, Ontario, Canada).

### 2.4. Histology

Whole hearts were excised from anesthetized mice, fixed in 4% paraformaldehyde, dehydrated through increasing concentrations of ethanol, and then embedded in paraffin. Heart sections (5 μm) were deparaffinized with xylene and rehydrated with consecutive incubations in decreasing concentrations of ethanol before incubation in distilled water. Sections were stained with Masson trichrome (Sigma-Aldrich, St. Louis, MO, USA) and imaged at 20× magnification using a Nikon AZ100 microscope (Tokyo, Japan) and NIS Elements software (Tokyo, Japan). Cardiomyocytes cross-sectional area was quantified with the NIS Elements. Quantification of fibrosis was performed with ImageJ software (NIH, Bethesda, MD, USA). All analyses were performed by an observer blinded to the conditions.

### 2.5. Oral Glucose Tolerance Test

Oral glucose tolerance test was performed after 6 h of fasting. The first glucose measurement was recorded at the end of 6 h prior to glucose administration (0-time point). After administration of glucose orally at the dose of 2 g/kg body weight, subsequent blood glucose measurements were taken at 15, 30, 45, 60, 90, and 120 min using a glucometer (Freedom Freestyle Lite; Abbott laboratories, Chicago, IL, USA).

### 2.6. Insulin Tolerance Test

Regular human insulin (Novo Nordisk, Princeton NJ, USA) was injected intraperitoneally (0.5 U/kg body weight). The first glucose measurement was recorded at the end of 6 h prior to insulin administration (0-time point). After the administration of insulin intraperitoneally, blood glucose measurements were taken at 15, 30, 45, 60, 90, and 120 min using a glucometer.

### 2.7. Sample Preparation and Immunoblotting

Tissue lysates were prepared from hearts as described previously [20]. Protein concentrations of lysates were quantified using the Bicinchoninic acid (BCA) protein assay (#23225 from Pierce, Waltham, MA, USA). Equal amounts of proteins were subjected to 4–20% gradient SDS-PAGE and subsequently transferred to PVDF membrane (Immobilon-P # IPVH00010, EMD Millipore, Danvers, MA, USA). Membranes were blocked for 2 h using LI-COR Odyssey blocking buffer (Lincoln, NE, USA), followed by incubation in primary antibody. Primary antibody incubations were performed at different dilutions for different antibodies, as described in the antibody list (in Supplementary data). All incubations for primary antibodies were performed overnight at 4 °C and followed by secondary antibody (IRDye 680LT or IRDye 800CW from LI-COR, Lincoln, NE, USA)) incubation at 1:4000 dilutions for 1 h at room temperature. After three washes (5 min each), blots were imaged with the Odyssey Infrared Imaging System (LI-COR, Lincoln, NE, USA)) and analyzed using the Image Studio Software (LI-COR, Lincoln, NE, USA)).

### 2.8. Antibodies

A detailed list of antibodies used for immunoblotting is provided in Table S2.

### 2.9. Statistical Analysis

Data are expressed as mean ± SEM. Differences between two groups were analyzed using an unpaired t-test (Graph Pad Prism Software Inc., San Diego, CA, USA). To analyze differences between the four groups (effect of diet and genotype) we used 2-way ANOVA followed by Tukey test for post hoc analysis using SigmaStat (Version 11, 2008, Systat Software, Inc., Chicago, IL, USA). Significance was accepted at *p* < 0.05.

## 3. Results

**Figure 1:**
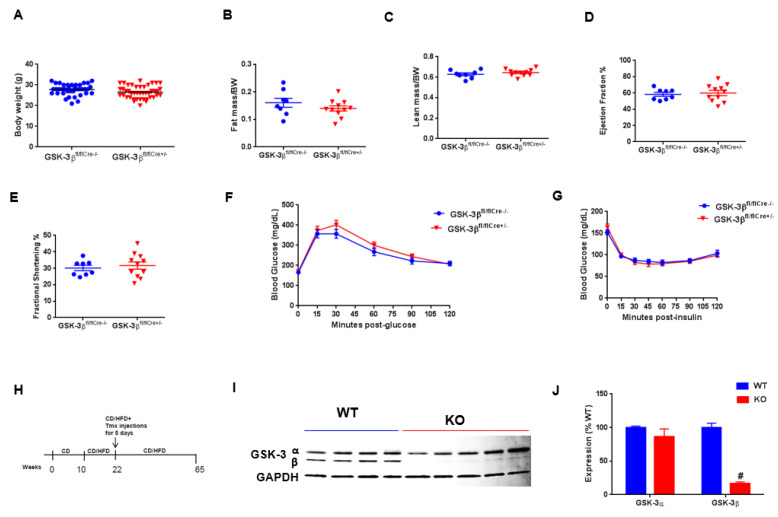
Characterization of cardiomyocyte (CM)-specific inducible Glycogen Synthase Kinase-3 beta (GSK-3β) KO mouse model. (**A**) Baseline body weights, (**B**) fat mass, (**C**) lean mass, (**D**) left ventricular ejection fraction (LVEF%), (**E**) left ventricular fractional shortening (LVFS%), (**F**) glucose tolerance (*n* = 13–14 per group), (**G**) insulin tolerance in GSK-3β^fl/flCre−/−^ and GSK-3β^fl/fl/Cre+/−^ animals (*n* = 8–11 per group), (**H**) experimental design. Ten-week-old male mice were fed CD or HFD diet for twelve weeks. After 12 weeks of control diet (CD) or high-fat diet (HFD), all mice received tamoxifen injections (20mg/kg/day, intraperitoneal injection, IP) for 5 consecutive days to delete GSK-3β specifically in CMs, and continued on CD or HFD for a total period of 55 weeks. (**I**) Immunoblot showing specific deletion of GSK-3β and (**J**) quantification of GSK-3α/β expression from wild type (WT) and KO hearts shows significant reduction in GSK-3β (~85%) expression in KO LV lysates compared to WT. As expected, GSK-3α expression was comparable between the WT and CM-GSK-3β KO hearts. ^#^*p* < 0.05 WT vs. KO.

**Figure 2:**
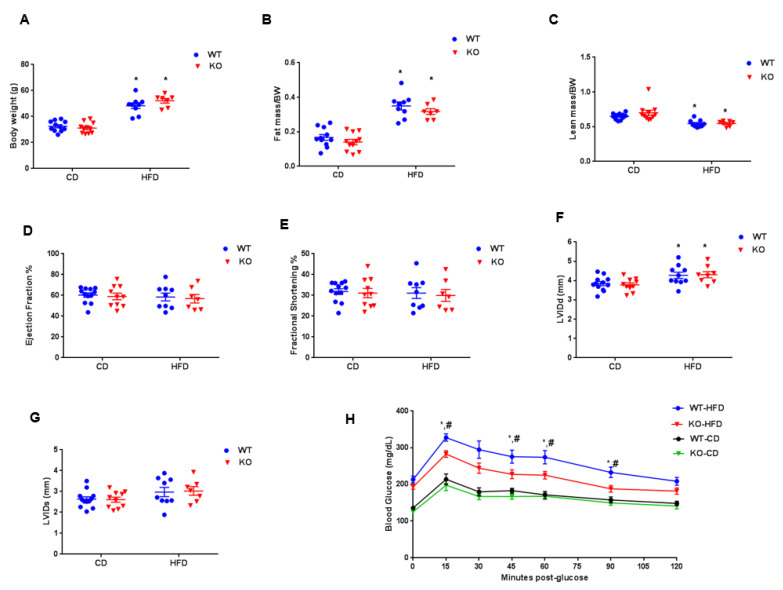
Cardiometabolic phenotyping of WT and CM-GSK-3β KOs post-HFD. (**A**) Body weights, (**B**) fat mass, (**C**) lean mass, (**D**) left ventricular ejection fraction, (**E**) left ventricular fractional shortening (**F**) left ventricular end-diastolic interior dimension (LVIDd), (**G**) left ventricular end-systolic interior dimension (LVIDs), (**H**) glucose tolerance test (GTT) in WT and KO animals after 55 weeks on CD or HFD (*n* = 10–12 per group). *p < 0.05 CD vs. HFD; ^#^*p* < 0.05 WT vs. KO.

CM-GSK-3β KO mice were generated using the tamoxifen-inducible Mer-Cre-Mer system to delete GSK-3β from fully mature cardiomyocytes [6]. At baseline, the body weight, cardiac function, fat, and lean masses were all comparable between the GSK-3β^fl/flCre−/−^ (WT) and GSK-3β^fl/flCre+/−^ (KO) groups (Figure 1A–E). These findings are consistent with our previous reports [6,10]. At baseline, glucose tolerance and insulin sensitivity were also similar between the GSK-3β^fl/flCre−/−^ and GSK-3β^fl/flCre+/−^ animals (Figure 1F,G). These results confirm that there was no effect of ‘Cre’ or “Lox P” insertion on the cardiometabolic profile. Tamoxifen treatment reduced the expression of CM-GSK-3β by 85% in the KO hearts compared to WT hearts (Figure 1H–J). Importantly, GSK-3α expression was comparable between the two groups after tamoxifen treatment (Figure 1I,J). As anticipated, high fat feeding led to a significant increase in body weights and fat mass in WT and KO animals (Figure 2A,B). HF-fed WT and KO animals had significantly lower lean mass compared to their controls on CD (Figure 2C). These findings are consistent with our previous report [6] and suggest that CM-GSK-3β inhibition does not affect HFD-induced body weight gain.

To determine the effect of GSK-3β deletion on cardiac function in an established obesity model, we performed two-dimensional echocardiography in WT and KO animals fed either a CD or HFD (Figure 2D,E). In spite of marked obesity, cardiac function (LV EF and LV FS) of WT mice fed HFD was similar to their littermates on CD (LV EF, WT CD: 60.3 ± 2.1, WT HFD: 58.5 ± 3.8; LV FS, WT CD: 31.8 ± 1.4, WT HFD: 31.1 ± 2.6). LV EF and LV FS were also comparable between the KO mice fed either a CD or HFD. However, left ventricular internal dimensions at end-diastole (LVIDd) were increased in both genotypes with HFD (Figure 2F,G). These findings are in stark contrast to our previous report [6] and suggest that the deletion of CM-GSK-3β after establishing the obesity does not lead to any adverse cardiac phenotype. To our complete surprise, CM GSK-3β deletion in the established obesity model improved systemic glucose tolerance in HF-fed KO animals compared to the WT (Figure 2H). This is an important finding, which warrants further investigation. It′s reported that conditional deletion of GSK-3β from skeletal muscle leads to improved glucose tolerance and insulin sensitivity [15]. However, herein, the GSK-3α/β levels in skeleton muscle of control and KO were comparable (Figure S1), suggesting that the observed improved glucose tolerance in HF-fed CM-GSK-3β KO is not due to a leaky deletion in the skeleton muscle.

**Figure 3:**
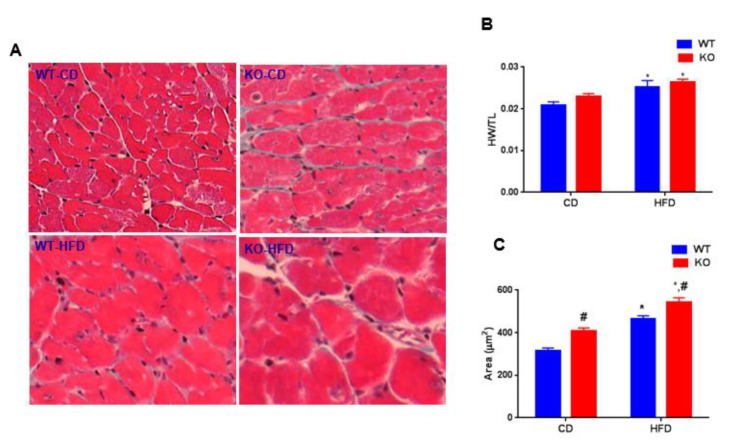
HFD-induced cardiac remodeling in WT and CM-GSK-3β –KOs. (**A**) HF feeding increased cardiac hypertrophy in WT and KO as seen with increased heart weight (HW)/tibia length (TL) (*n* = 7–11 per group). (**B**) Representative Masson trichrome-stained sections of whole heart isolated from WT and CM-GSK-3β KO animals at 55 weeks post CD or HFD. (**C**) Quantification of CM cross-sectional area at 55 weeks post CD or HFD from WT and KO animals (*n* = 6–8 per group). *p < 0.05 CD vs. HFD; ^#^*p* < 0.05 WT vs. KO.

To determine the role of GSK-3β on cardiac remodeling, we calculated the ratio of heart weight (HW) to tibia length (TL) in WT and CM-GSK-3β KO animals fed either a CD or HFD. High-fat feeding significantly increased HW/TL ratio in both WT and KO animals (Figure 3A). To examine hypertrophy at the cellular level, CM cross-sectional areas were determined (Figure 3B,C). Consistent with our previous reports, CM cross-sectional area was increased in KO hearts compared to WT fed CD. As expected, HFD increased the CM area in both the genotypes. Of note, CM cross-sectional area was significantly higher in HF-fed KO animals compared to the WT. Consistently, Q-PCR analysis revealed a clear trend of increased ANP and brain natriuretic peptide (BNP) expression in the HFD-fed KOs (statistically not significant) (Figure S2). Taken together, these results suggest that deleting CM-GSK-3β in obese does lead to cardiac hypertrophy.

**Figure 4:**
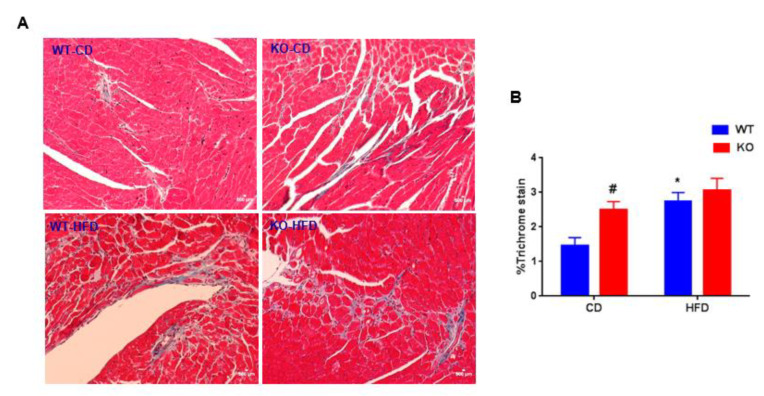
HFD increases fibrosis in WT and KO hearts: (**A**) Representative images of the left ventricle (LV) in WT and KO hearts stained with Masson trichrome 55 weeks post CD or HFD, (**B**) percent fibrosis was determined from 6–8 random images taken from six animals from each group. *p < 0.05 CD vs. HFD; ^#^*p* < 0.05 WT vs. KO.

**Figure 5:**
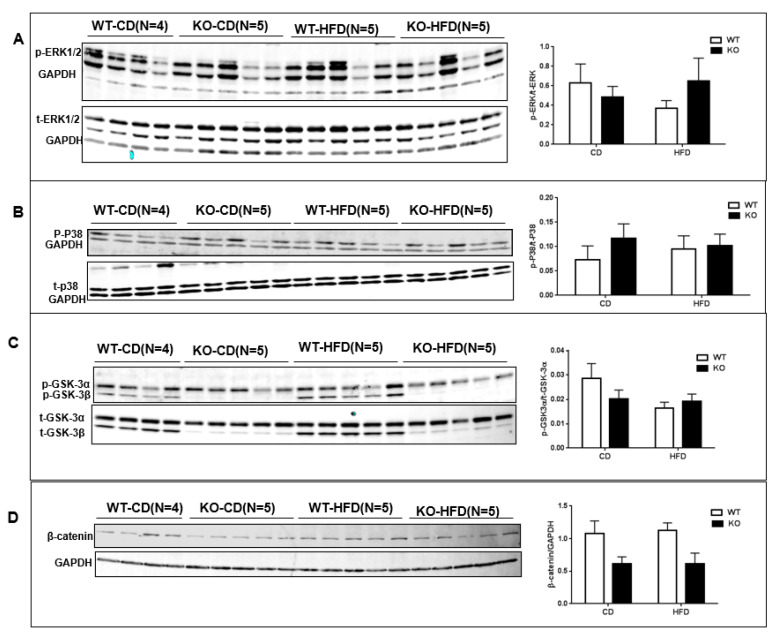
Analysis of signaling pathways in WT and CM-GSK-3β–Kos. LV lysates from WT and CM-GSK-3β KO animals were analyzed by immunoblotting with the following antibodies: (**A**) ERK phosphorylated at Thr202/Tyr204, (**B**) p38 phosphorylated at Thr180/Tyr182, (**C**) total and phospho-GSK-3α/β, (**D**) β-catenin expression in WT and KO hearts fed CD or HFD for 55 weeks.

Excessive fibrotic remodeling is another hallmark feature of adverse cardiac remodeling [8]. To evaluate the pathological cardiac remodeling, cardiac fibrosis was measured in WT and CM-GSK-3β KO animals fed either a CD or HFD using Masson trichrome staining. CM-GSK-3β deletion increased cardiac fibrosis on CD (Figure 4A,B). In agreement with previous reports, the percent of cardiac fibrosis was higher in the HF-fed WT compared to the littermate controls on CD [21]. However, we did not see an additive effect of CM-GSK-3β deletion on HFD mediated myocardial fibrosis (Figure 4A,B). Next, we sought to investigate the molecular mechanisms responsible for the differential cardiac phenotype in CM-GSK-3β KOs subjected to developmental versus established obesity. Mitogen-activated protein kinases (MAPKs), such as extracellular signal-regulated kinases (ERKs), P38, and c-Jun N-terminal kinases (JNKs) which have been shown to be altered with high-fat feeding and are critical to cardiac pathophysiology, were unaltered (Figure 5A,B). Previously, in the developing obesity model, we saw a reduction in cardiac GSK-3α activity (decreased GSK-3α phosphorylation) in HF-fed CM-GSK-3β KO hearts leading to an impairment in GSK-3-β-catenin degradation pathway and cardiac dysfunction [6]. Interestingly, herein, we observed a comparable phospho-GSK-3α (activity) and β-catenin levels between the KO and control hearts (Figure 5C,D). These results indicate that in an established obesity model, GSK-3α is sufficient to maintain the efficient signaling for β-catenin ubiquitination.

## 4. Discussion

Herein, we report that CM-specific deletion of GSK-3β in obese animals leads to improved systemic glucose tolerance. Furthermore, we demonstrate that obese CM-GSK-3β KO maintains the normal cardiac function and does not display adverse cardiac phenotype of excessive fibrosis and adverse remodeling. Improved systemic glucose tolerance in CM-specific GSK-3β KO animals emphasizes the contribution of myocardium in global glucose metabolism. This exciting observation is consistent with several recent reports that heart can influence systemic metabolism [4,5,22,23]. Indeed, numerous reports support the hypothesis that pharmacological inhibition or genetic deletion of GSK-3β in various organs improves systemic glucose homeostasis [15,17,18,24,25]. This hypothesis is further supported by numerous studies showing increased GSK-3β activity in type 2 diabetes patients [26,27,28].

In the present study, we observed a comparable cardiac function and ventricular remodeling in the CM-GSK-3β KOs and control hearts. However, these findings are in stark contrast to our previous report with developing obesity model showing modest but significant cardiac dysfunction in HF-fed CM-GSK-3β KOs [6]. These differential phenotypic outcomes with developmental vs. established obesity models are important findings and warrants further investigation. Having that said, multiple studies have demonstrated a context-specific role of GSK-3 isoforms in cardiac metabolism and function [7,10,11,12,29,30]. We have reported [10] the stress-specific role of GSK-3β on cardiac hypertrophy. Specifically, deletion of CM-GSK-3β led to physiological hypertrophy after ischemic injury; however, it does not regulate pressure overload-induced cardiac hypertrophy. We have also reported [12] that cardiac fibroblast (CF)-specific deletion of GSK-3β leads to pathological cardiac hypertrophy in ischemic hearts. Furthermore, aberrant accumulation of β-catenin is known to negatively regulate the cardiomyocyte size [31]. We observed a differential effect of GSK-3β deletion on the β-catenin accumulation in developing vs. established obesity, which may account for the variable hypertrophic response [6]. Taken together, these studies suggest that GSK-3β mediated regulation of cardiac hypertrophy is specific to the employed stress, as well as to cell-specific gene targeting.

Signaling cascades, such as ERKS, P38, and JNKs have been implicated in high-fat feeding-induced obesity and are crucial to cardiac pathophysiology. In the present study, we did not observe any aberrant activation of these signaling pathways. We speculate that the differential observation regarding these pathways in our study vs. published literature might relate to the temporal effect of HFD treatment and the dynamic nature of signaling cascades. Indeed, in the present study, we have chosen a genuinely chronic setting (55 weeks of HFD); however, most of the studies in the literature have focused on comparatively acute setting. Previously, we have shown that on a CD, GSK-3α compensates for the loss of CM-GSK-3β to prevent the accumulation of its downstream target β-catenin, which is strongly associated with various cardiac pathologies [6]. We have also shown that this protective compensatory mechanism is lost in developing obesity, leading to excessive accumulation of β-catenin and cardiac dysfunction. Herein, we did not observe the aberrant accumulation of β-catenin in CM-GSK-3β KO’s hearts. Consistently, phospho-GSK-3α (activity) was unaltered between the KO hearts on either diet. These findings indicate that in established obesity, GSK-3α compensate for the loss of GSK-3β, and facilitate the efficient signaling for β-catenin ubiquitination. This observation is consistent with previous reports showing deletion of both alleles of GSK-3β was insufficient to lead to the stabilization of β-catenin and, therefore, to activate the canonical Wnt signaling [32]. In fact, deletion of at least three alleles of GSK-3 was required to begin any increase in the cellular β-catenin levels [32]. These findings are also supported by the fact that modest GSK-3 inhibitor lithium has been used for many years to treat patients with bipolar disorder without any significant adverse cardiac effects. Although there are several ongoing clinical trials to test the efficacy of GSK-3 inhibition on a wide variety of medical conditions, to our knowledge, there is no specific trial with GSK-3 inhibitor in average weight vs. obese subjects. Our findings suggest that the cardiac function of such patients should be closely monitored. In reference to the future perspective, the molecular basis for the differential effect of developing vs. established obesity on GSK-3α phosphorylation and β-catenin signaling is interesting, and needs further investigation.

## Supplementary Materials

The following are available online at https://www.mdpi.com/2073-4409/9/5/1120/s1, Table S1. TaqMan gene expression assays used for the quantification of fetal gene program. Table S2. Detailed list of different antibodies used and applications, Figure S1. Comparable expression of GSK-3α/β in the skeleton muscle of controls and CM-GSK-3β Kos, Figure S2. A strong trend of fetal gene program activation in high-fat fed CM-GSK-3β KOs.

## Abbreviations

ANP	Atrial natriuretic peptide
BNP	Brain natriuretic peptide
CD	Control diet
CF	Cardiac fibroblast
CM	Cardiomyocyte
CVD	Cardiovascular diseases
EF	Ejection fraction
FS	Fractional shortening
GSK-3	Glycogen Synthase Kinase-3
HFD	High-fat diet
HW	Heart weight
IP	Intraperitoneal injection
LVIDd	LV interior dimension -diastolic
LVIDs	LV interior dimension -systolic
MI	Myocardial infarction
TAC	Transverse Aortic Constriction
TL	Tibia length
