# Supplementary material for: Deletion of Cardiomyocyte Glycogen Synthase Kinase-3 Beta (GSK-3β) Improves Systemic Glucose Tolerance with Maintained Heart Function in Established Obesity
Source: Cells. 2020 Apr 30;9(5):1120. doi: 10.3390/cells9051120 (PMC7291092; doi:10.3390/cells9051120)
Supplement: Supplementary file 1 [file cells-09-01120-s001.pdf]

Supplementary Material

Deletion of Cardiomyocyte GSK-3β Improves Systemic Glucose Tolerance with Maintained Heart Function in Established Obesity

Manisha Gupte [1,2], Prachi Umbarkar [1,3], Anand Prakash Singh [1,3], Qinkun Zhang [1,3], Sultan Tousif [3] and Hind Lal [1,3,*]

1. Division of Cardiovascular Medicine, Vanderbilt University Medical Center, Nashville, 37232, TN, USA; guptem@apsu.edu (M.G.); pumbarkar@uabmc.edu (P.U.); apsingh@uabmc.edu (A.P.S.); qzhang@uabmc.edu (Q.Z.)
2. Department of Biology, Austin Peay State University, Clarksville, 37044, TN, USA
3. Division of Cardiovascular Disease, UAB | University of Alabama at Birmingham, Birmingham, 35294-1913, AL, USA; sana80@uab.edu

* Correspondence: hindlal@uabmc.edu; Tel: (205) 996-4219; Fax: (205) 975-5104

RNA Extraction and Quantitative PCR Analysis

Total RNA was extracted from heart tissue using the RNeasy Mini Kit (74104, Qiagen, Hilden, Germany) according to manufacturer's protocol. cDNA was synthesized using the iScript cDNA synthesis kit (170-8891, Bio-Rad Laboratories, Hercules, CA, USA) following manufacturer's instructions. Gene expression was analyzed by quantitative PCR (qPCR) using the TaqMan Gene Expression Master Mix (4369016, Applied Biosystems, Foster City, CA, USA) and TaqMan gene expression assays as specified in the table immediately below. The assay was performed with a Bio-Rad CFX96 Real-Time PCR Detection machine (Bio-Rad Laboratories, Hercules, CA, USA) . Relative gene expression was determined by using the comparative C_T method ($2^{-\Delta\Delta C_T}$) and was represented as fold change. Briefly, the first ΔC_T is the difference in threshold cycle between the target and reference genes: $\Delta C_T = C_T$ (a target gene X) − C_T (18SrRNA) while $\Delta\Delta C_T$ is the difference in ΔC_T as described in the above formula between the CTL and KO group, which is = ΔC_T (KO target gene X) − ΔCT (CTL target gene X). Fold change is calculated using $2^{-\Delta\Delta C_T}$ equation.

Table S1. TaqMan gene expression assays used for the quantification of fetal gene program.

Gene	Assay ID	Cat. No.
Nppa (ANP)	Mm01255747_g1	4331182
Nppb (BNP)	Mm01255770_g1	4331182
Eukaryotic 18S rRNA Endogenous Control		4319413E

Table S2. Detailed list of different antibodies used and applications.

S. No.	Antibody	Vendor	Catalog No	Dilution	Application
1	Beta-Catenin	Cell Signaling	9562	1:1000	Western Blotting
2	phospho-GSK-3α/β(Ser21/9)	Cell Signaling	9331	1:1000	Western Blotting
3	GAPDH	Fitzgerald	10R-G109a	1:10,000	Western Blotting
4	GSK-3α/β	Cell Signaling	5676	1:1000	Western Blotting
5	p-ERK	Cell Signaling	4370	1:1000	Western Blotting
6	T-ERK	Santacruz	93	1:1000	Western Blotting
7	p-JNK	Cell Signaling	9255	1:1000	Western Blotting
8	t-JNK	Cell Signaling	9252	1:1000	Western Blotting
9	p-p38	Cell Signaling	9211	1:1000	Western Blotting
10	P38	Santacruz	535	1:1000	Western Blotting
11	p-AKT	Cell Signaling	4060	1:1000	Western Blotting
12	t-AKT	Cell Signaling	4691	1:1000	Western Blotting

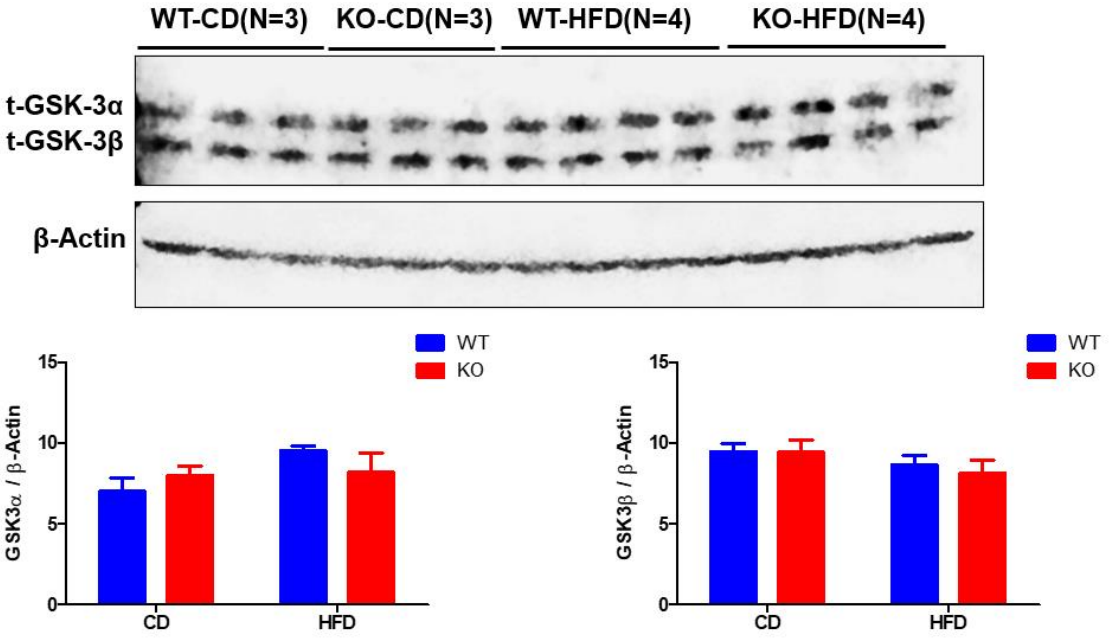

Figure S1. Comparable expression of GSK-3α/β in the skeleton muscle of controls and CM-GSK-3β KOs.

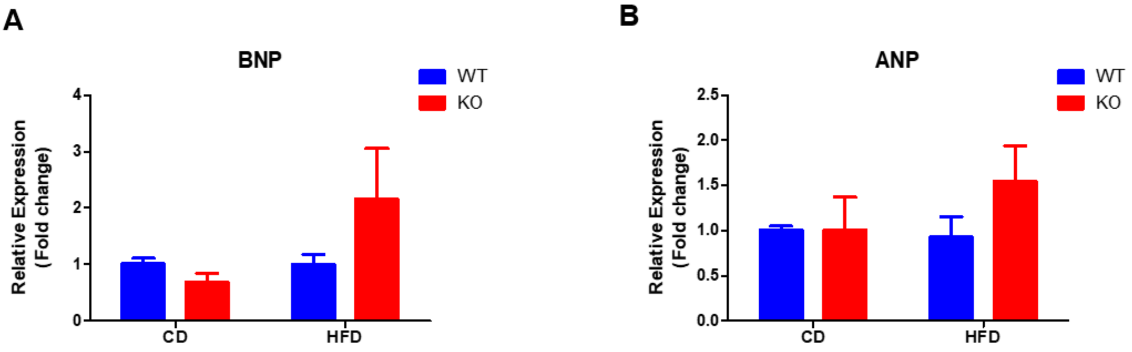

Figure S2. A strong trend of fetal gene program activation in high fat fed CM-GSK-3β KOs. CD–WT, N = 3; CD–KO, N = 5; HFD–WT, N = 6; HFD–KO, N = 5.
